# Inflammation drives pathogenesis of early intestinal failure-associated liver disease

**DOI:** 10.1038/s41598-024-54675-9

**Published:** 2024-02-20

**Authors:** Scott C. Fligor, Savas T. Tsikis, Thomas I. Hirsch, Ashish Jain, Liang Sun, Shira Rockowitz, Kathleen M. Gura, Mark Puder

**Affiliations:** 1 Vascular Biology Program and Department of Surgery, Boston Children’s Hospital, Boston, MA 02115 USA; 2 Harvard Medical School, Boston, MA USA; 3 Research Computing, Information Technology, Boston Children’s Hospital, Boston, MA USA; 4 Division of Genetics and Genomics, and the Manton Center for Orphan Disease Research, Boston Children’s Hospital, Boston, MA USA; 5 Department of Pharmacy and the Division of Gastroenterology and Nutrition, Boston Children’s Hospital, Boston, MA USA

**Keywords:** Liver diseases, Gastrointestinal models, Nutrition disorders, RNA sequencing

## Abstract

Patients with intestinal failure who receive long-term parenteral nutrition (PN) often develop intestinal failure-associated liver disease (IFALD). Although there are identified risk factors, the early pathogenesis is poorly understood and treatment options are limited. Here, we perform a transcriptomic analysis of liver tissue in a large animal IFALD model to generate mechanistic insights and identify therapeutic targets. Preterm Yorkshire piglets were provided PN or bottle-fed with sow-milk replacer for 14 days. Compared to bottle-fed controls, piglets receiving PN developed biochemical cholestasis by day of life 15 (total bilirubin 0.2 vs. 2.9 mg/dL, *P* = 0.01). RNA-Seq of liver tissue was performed. Ingenuity Pathway Analysis identified 747 differentially expressed genes (343 upregulated and 404 downregulated) with an adjusted *P* < 0.05 and a fold-change of > |1|. Enriched canonical pathways were identified, demonstrating broad activation of inflammatory pathways and inhibition of cell cycle progression. Potential therapeutics including infliximab, glucocorticoids, statins, and obeticholic acid were identified as predicted upstream master regulators that may reverse the PN-induced gene dysregulation. The early driver of IFALD in neonates may be inflammation with an immature liver; identified therapeutics that target the inflammatory response in the liver should be investigated as potential treatments.

## Introduction

Intestinal failure occurs when the intestinal length or function is insufficient to absorb nutrients or fluids through the gastrointestinal tract. In children, intestinal failure most often occurs following extensive bowel resection for necrotizing enterocolitis, malrotation with volvulus, or congenital anomalies including intestinal atresia^1^. Survival depends upon long-term parenteral (intravenous) nutrition to support growth and development, but long-term parenteral nutrition (PN) often leads to progressive cholestatic liver disease known as intestinal failure-associated liver disease (IFALD)^2^. In recent years, advances in therapy for IFALD–such as fish oil-containing lipid emulsions and multidisciplinary intestinal rehabilitation programs—have reduced the need for liver or intestinal transplant, and survival is now possible for decades^3^. However, even with modern management, most children with intestinal failure on long-term PN will have elevated liver enzymes and abnormal histology on routine liver biopsy, with some demonstrating progressive fibrosis^4^. New therapies are needed for IFALD.

The pathogenesis of IFALD is multifactorial and not fully elucidated. Numerous risk factors have been implicated including prematurity, low birth weight, long-term PN, lack of enteral nutrition, sepsis, surgical procedures, disrupted intestinal barrier, small bowel bacterial overgrowth/dysbiosis, and omega-6 fatty acid rich lipid emulsions high in phytosterols^5^. However, the early mechanisms driving the development of IFALD remain unclear and nearly all mechanistic studies have occurred in small animal models^6,7^. In a human study of infants with IFALD, RNA Sequencing (RNA-Seq) was performed on plasma cell free RNA/microRNA from four patients at the initiation of fish oil lipid emulsion therapy and at six months^8^. After six months of therapy, microRNA 122, reactive oxygen species, and various inflammatory pathways were downregulated compared to baseline. To our knowledge, no large animal or human studies have investigated the transcriptome in liver tissue to elucidate early mechanisms of IFALD and identify potential therapeutic strategies.

Human liver tissue is not readily available to investigate the transcriptomic changes in early IFALD. Diagnosis is made clinically based upon biochemical markers of cholestatic liver injury, including direct/total bilirubin and gamma glutamyl transferase. Liver biopsy is not routinely performed as it is not necessary for diagnosis and does not alter initial management^9^. We have recently utilized a preterm piglet model of IFALD that is similar to human neonatal IFALD, both in pathogenesis and in outcomes^10^. Preterm piglets were provided PN for two weeks, resulting in cholestatic and steatotic liver disease. RNA-Seq was performed on liver tissue obtained from this study and compared to liver tissue from a second cohort of age-matched preterm piglets that were bottle-fed with sow milk replacer. We then utilized Ingenuity Pathway Analysis to identify key pathways that are dysregulated in early IFALD and highlight potential therapeutics that counteract the gene dysregulation in a pilot, hypothesis-generating study.

## Results

### Parenteral nutrition treatment in preterm piglets results in biochemical cholestasis and hepatosteatosis

**Figure 1:**
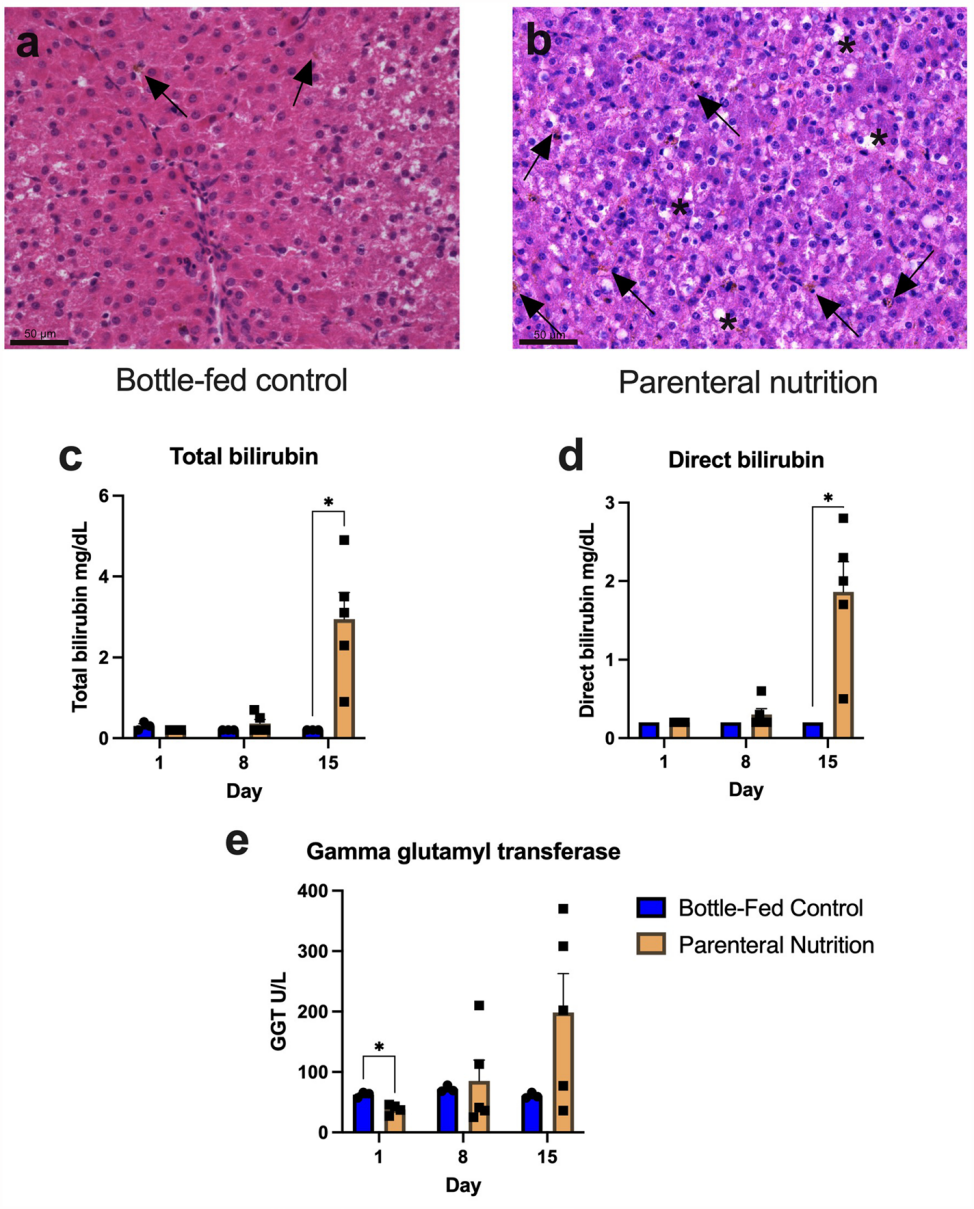
Parenteral nutrition in preterm piglets results in histologic and biochemical cholestasis by day of life 15. Representative hematoxylin & eosin-stained liver tissue is shown from a bottle-fed piglet (**a**) and a PN piglet (**b**). Piglets receiving PN demonstrate extensive bile deposition (arrows) and steatosis (asterisk), while the bottle-fed piglets have mild bile pigment predominantly noted within Kupfer cells (arrows), with rare steatosis. Piglets in the PN group developed biochemical cholestasis marked by increased plasma total bilirubin (**c**), direct bilirubin (**d**), and gamma glutamyl transferase (**e**) at day of life 15. Mean ± SEM. Comparisons with Welch’s t-test with Holm-Sidak correction for multiple testing. *P < 0.05.

All piglets in the PN group and bottle-fed group survived to day of life (DOL) 15. Liver histology was assessed by a masked veterinary pathologist. Representative formalin-fixed paraffin-embedded hematoxylin and eosin-stained liver tissue is shown in Fig. 1a and b. Piglets in the PN group demonstrated extensive bile pigment and steatosis, while piglets in the bottle-fed group demonstrated minimal bile pigment or steatosis. Biochemical markers of cholestatic liver injury were assessed at DOL 1, 8, and 15 (Fig. 1c−e). Compared to the bottle-fed control group, piglets in the PN group developed cholestatic liver injury at DOL 15 (assessed immediately prior to sacrifice) marked by elevated total bilirubin (0.2 vs. 2.9 mg/dL, *P* = 0.01), direct bilirubin (0.2 vs. 1.9 mg/dL, *P* = 0.01), and gamma glutamyl transferase (61 vs. 199 U/L, *P* = 0.10).

### Differentially expressed genes

**Figure 2:**
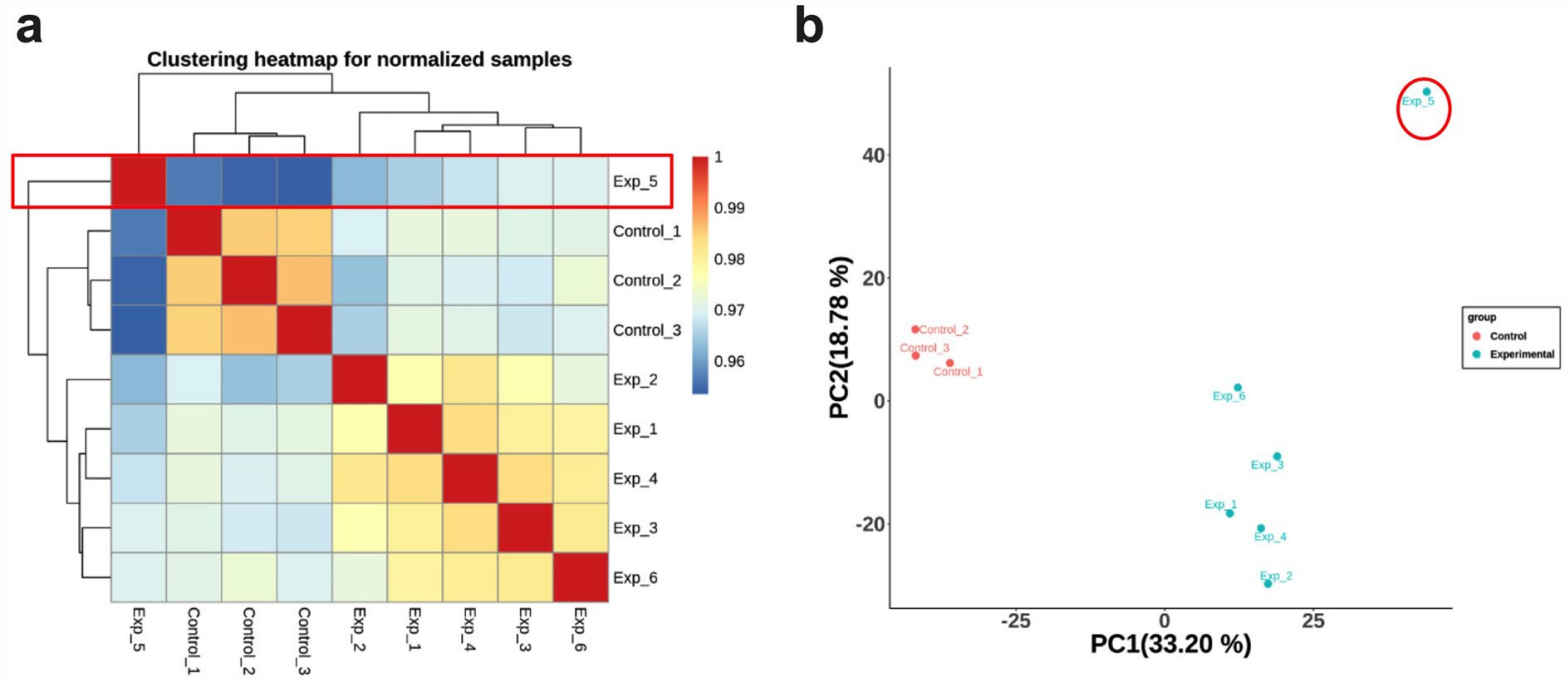
Clustering heatmap (**a**) and principal component analysis (**b**) of differential gene expression. One outlier from the PN group was excluded (circled) on the basis of substantial deviation from both the bottle-fed group and the remaining PN samples.

RNA-Seq was performed with the Illumina NovaSeq 6000 system on three bottle-fed control animals and six experimental animals that received two weeks of PN. All samples passed initial RNA extraction quality testing for a RNA integrity number > 4 prior to RNA-Seq. Following RNA-Seq and mapping to the reference pig genome, differentially expressed genes (DEGs) were identified. As part of the quality-control process, a principal component analysis and sample clustering using normalized data were performed to assess for reproducibility among biological replicates; one outlier was excluded from the PN group due to substantial deviation in gene expression from both the control and other experimental samples (Fig. 2).

Out of the 2060 identified DEGs (1064 upregulated, 996 downregulated), Ingenuity Pathway Analysis identified 747 molecules (343 upregulated and 404 downregulated) with an adjusted *P* < 0.05 and a log fold-change of > |1|, which were used for the subsequent pathway analysis. Most molecules were located in the cytoplasm (38%, n = 146 up, 139 down) followed by the plasma membrane (21%, n = 42 up, 117 down), nucleus (20%, n = 91 up, 57 down), extracellular space (11%, n = 33 up, 47 down), with the remainder classified as ‘other’.

There were 30 upregulated transmembrane receptors, largely involved in inflammatory signaling (gene, expression log ratio). These included tumor necrosis factor (TNF) receptors (TNFRSF18, 5.671; TNFRSF9, 3.908; TNFRSF21, 1.523), toll like receptor (TLR) 9 (TLR9, 1.969), CD72 (2.616), Fas cell surface death receptor (FAS, 2.740), and interferon receptors (IFNGR2, 1.509; IFNAR1, 1.464; IFNAR2, 1.205). Adhesion molecules were also upregulated (VCAM1, 1.521; CXADR, 1.416; ICAM1, 1.076; ITGB3, 1.152). CD200 receptor 1 was downregulated (CD200R1, − 2.855), Eleven cytokines were upregulated (gene, log ratio; LIF, 5.450; CXCL8, 4.539; CXCL10, 3.681; CXCL9, 3.297; WNT4, 2.779; CCL20, 2.286; SPP1, 2.020; CCL19, 1.739; TIMP1, 1.289; CLCF1, 1.101), compared to three which were downregulated (CXCL12, − 1.803; CXCL11, − 1.680; IL21, − 4.877).

### KEGG analysis

**Figure 3:**
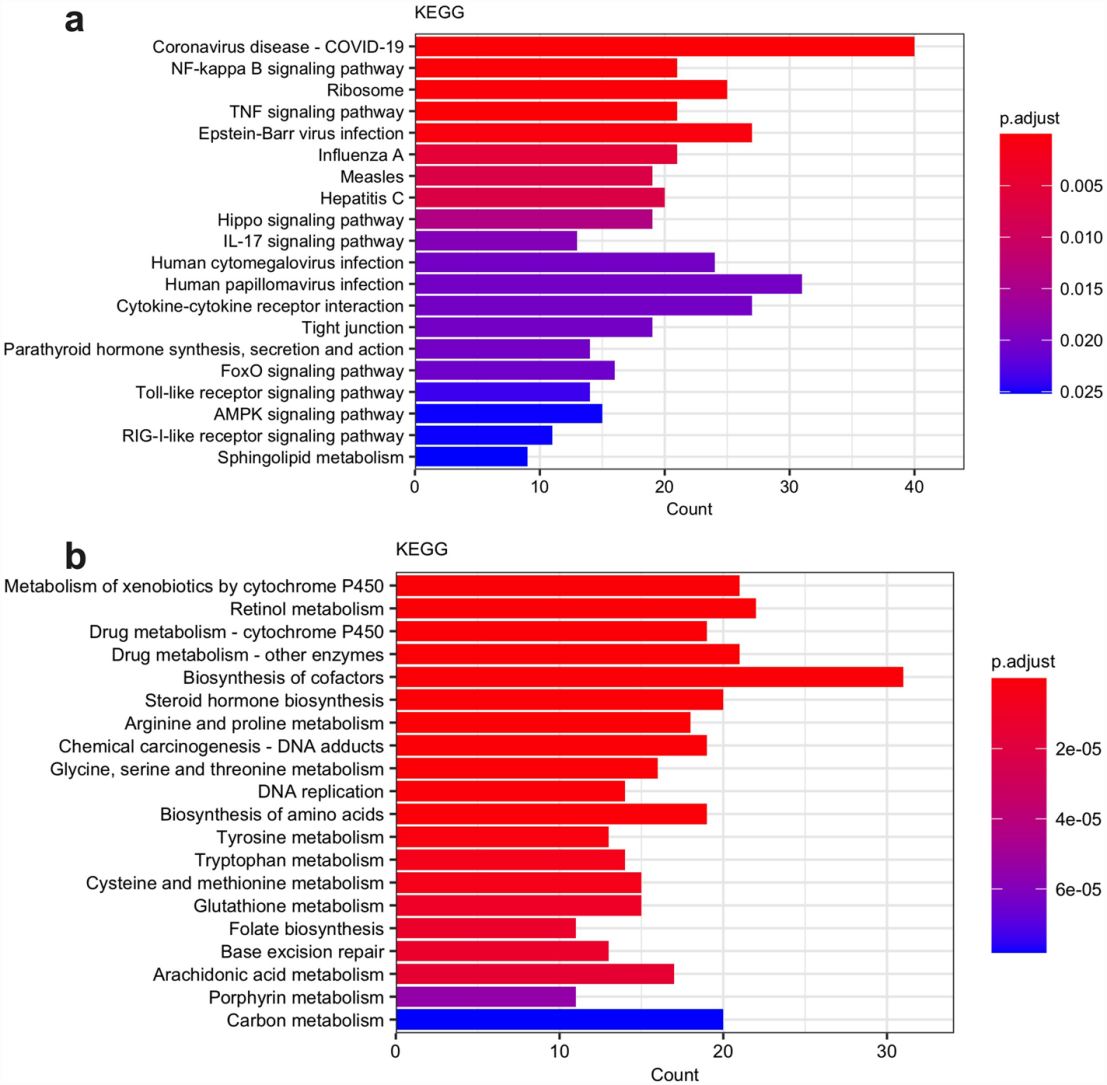
Top upregulated and downregulated Kyoto Encyclopedia of Genes and Genomes (KEGG) pathways. Compared to bottle-fed controls, top upregulated KEGG pathways in PN piglets include inflammatory signaling pathways (**a**), while top downregulated pathways are primarily involved in metabolism (**b**). The x-axis demonstrates the number of differentially expressed genes in a given pathway, while the color demonstrates the adjusted p-value. Enrichment analysis and figure created by clusterProfiler (v 4.6.2) R package.

An initial screen of enriched biological pathways was performed using the Kyoto Encyclopedia of Genes and Genomes (KEGG) database^11–13^. The top twenty down- and upregulated KEGG pathways determined in clusterProfiler are shown in Fig. 3^14^. Upregulated pathways (Fig. 3a) were largely related to inflammation or infection, including coronavirus disease, nuclear factor kappa B (NF-kB) signaling pathway, TNF signaling pathway, cytokine-cytokine receptor interaction, and toll-like receptor signaling pathway. Downregulated pathways were primarily related to metabolic processes (Fig. 3b).

### Canonical pathway analysis

**Figure 4:**
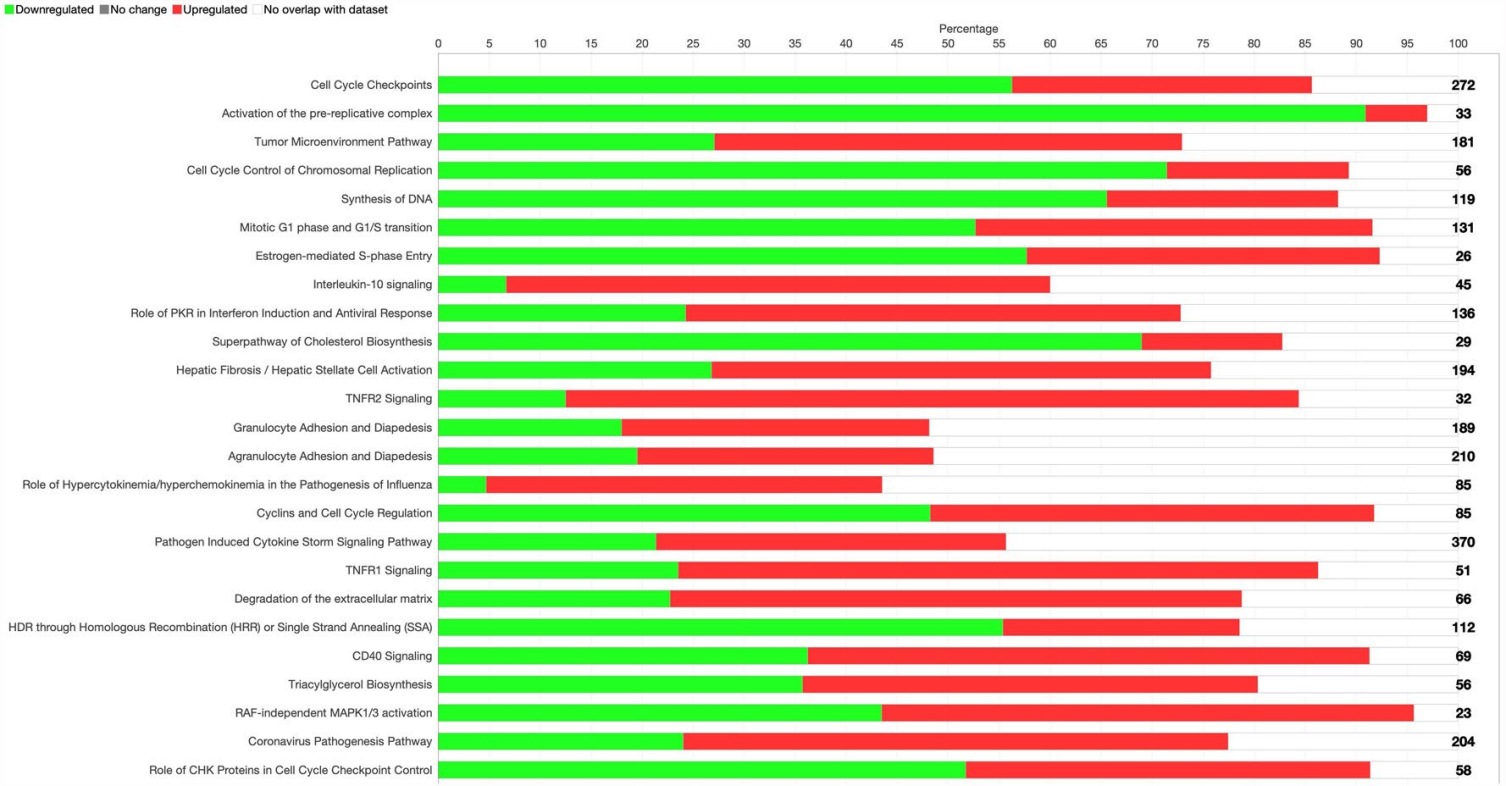
Top 25 enriched canonical pathways identified in Ingenuity Pathway Analysis. The number of total genes in the pathway are designated by the number to the right of the stacked horizontal bar graph. Compared to bottle-fed controls, the percentage of genes within the pathway that are downregulated in PN piglets (adjusted *P* < 0.05, log-fold change < − 1.0) are shown in green. The percentage of genes within the pathway that are upregulated (adjusted *P* < 0.05, log-fold change > 1.0) are shown in red. Genes that are neither up or downregulated are shown in white.

Ingenuity Pathway Analysis identified top enriched canonical pathways (Fig. 4). The key enriched pathways demonstrated alterations in cell cycle control and inflammation. Canonical pathways involved in cell cycle control had negative z scores, reflecting inhibited cell cycle progression (z-score, upregulated/total genes, downregulated/total genes). These pathways included cell cycle checkpoints (z-score: − 4.700, Up: 153/272, Down: 80/272); activation of the pre-replicative complex (z-score: − 3.464, Up: 2/33, Down: 30/33); cell cycle control of chromosomal replication (z-score: − 3.207, Up: 10/56, Down: 40/56); and synthesis of DNA (z-score: − 3.638, Up: 27/119; Down: 78/119).

**Figure 5:**
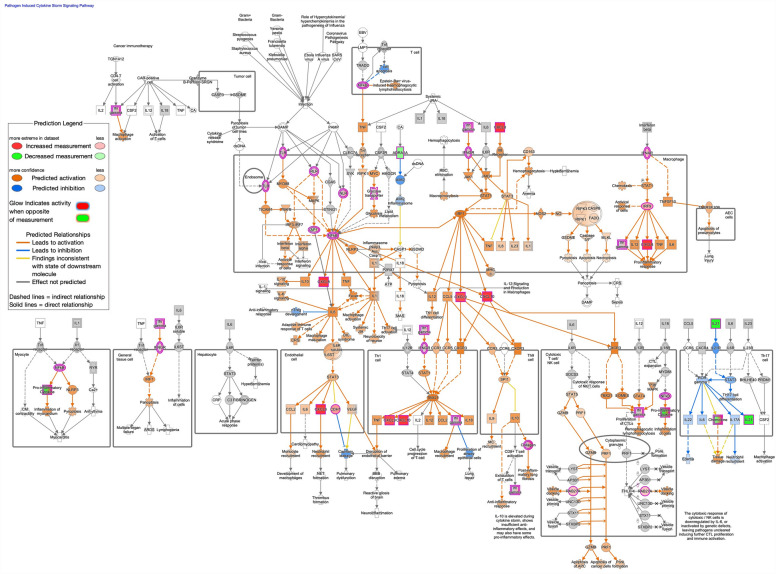
Predicted activation of pathogen induced cytokine storm signaling pathway in parenteral nutrition piglets mediated by NF-kB, STAT1, and STAT3. Pathway assessed in Ingenuity Pathway Analysis. Darker color demonstrates greater magnitude of pathway activation or inhibition.

**Figure 6:**
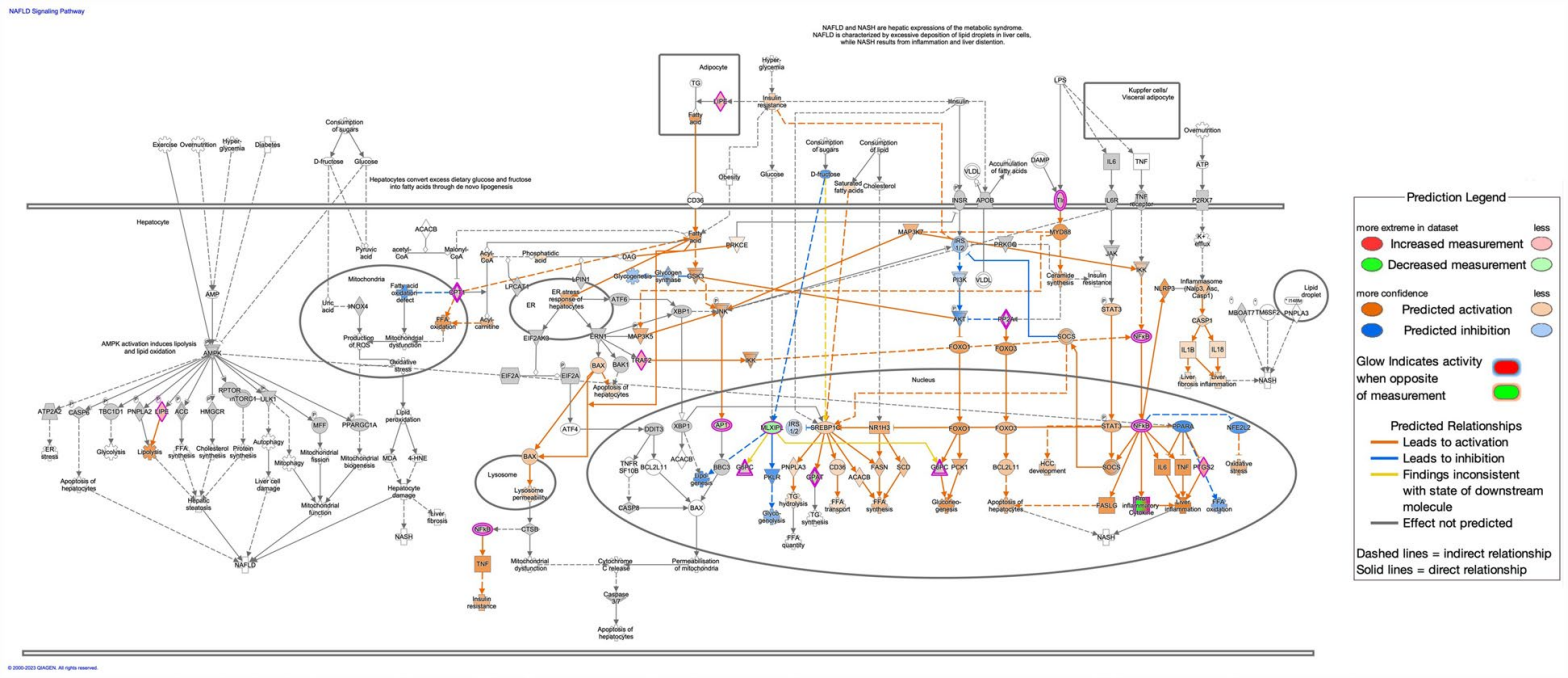
Predicted activation of NAFLD signaling pathway in parenteral nutrition piglets. Pathway assessed in Ingenuity Pathway Analysis. Darker color demonstrates greater magnitude of pathway activation or inhibition.

**Figure 7:**
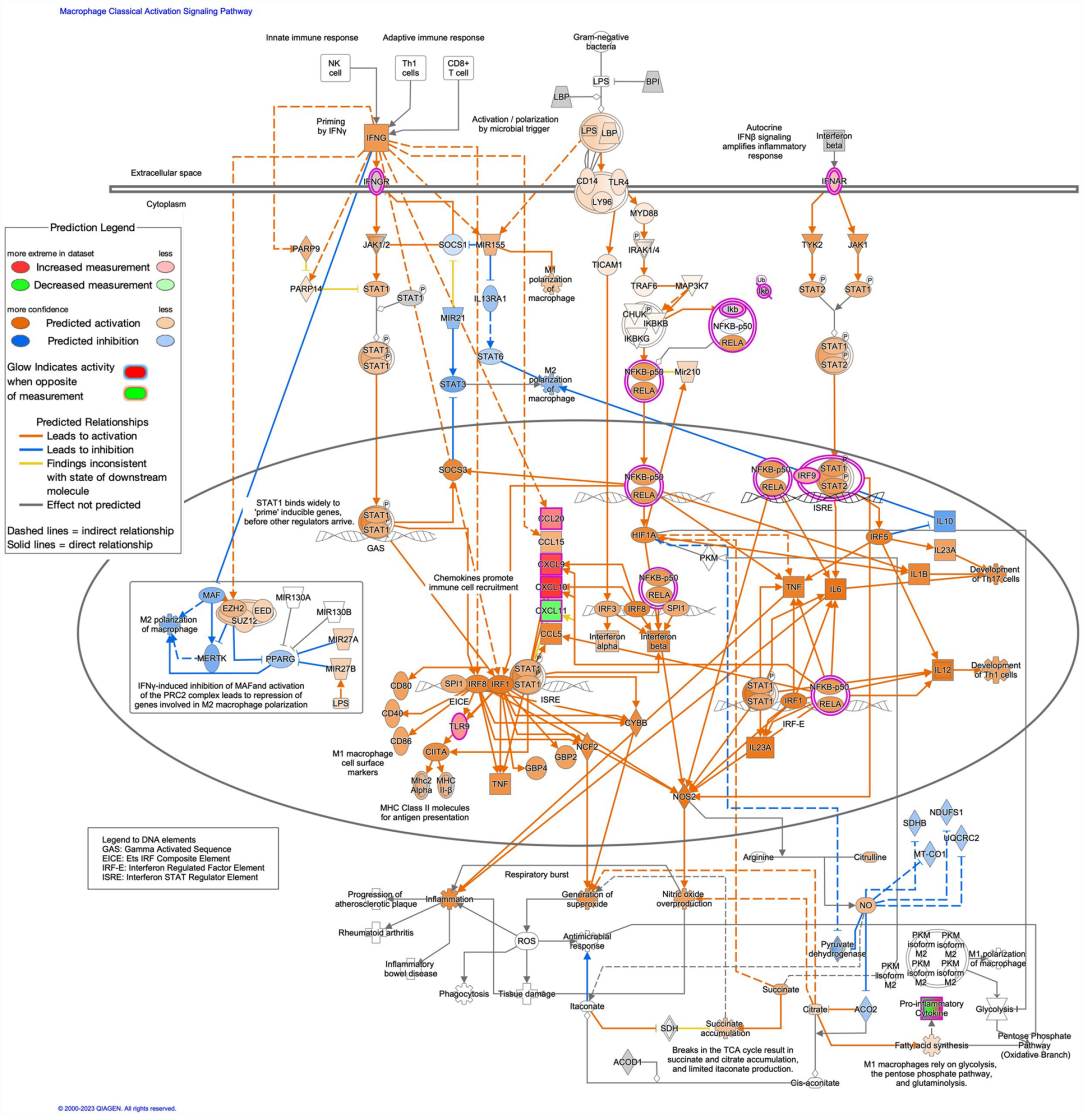
Predicted activation of macrophage classical activation signaling pathway in parenteral nutrition piglets. Pathway assessed in Ingenuity Pathway Analysis. Darker color demonstrates greater magnitude of pathway activation or inhibition.

Inflammation pathways were generally upregulated, including tumor microenvironment pathway (z-score: 2.858, Up: 83/181, Down: 49/181); interleukin-10 signaling (z-score: 3.162, Up: 24/45, Down: 3/45); pathogen induced cytokine storm signaling pathway (Fig. 5; z-score: 2.268, Up: 127/370, Down: 79/370); and CD40 signaling (z-score: 0.707, Up: 38/69, Down: 25/69). Notable additional enriched canonical pathways included S100 family signaling pathway (z-score: 3.507, Up: 249/773, Down: 202/773); nonalcoholic fatty liver disease (NAFLD) signaling pathway (Fig. 6, z-score: 1.886, Up: 109/226, Down: 56/226); and macrophage classical activation signaling pathway (Fig. 7, z-score: 1.941, Up: 75/188, Down: 40/188).

### Upstream regulator analysis

Upstream regulator analysis identifies potential upstream regulators that may explain downstream changes in the observed dataset with an adjusted *P* < 0.05. The top twenty upstream regulators (excluding drugs and chemicals) with the highest (or lowest) activation Z scores are shown in Table 1. Most of the top activated upstream regulators (i.e., determined by a positive Z score) are core mediators of the inflammatory response and immunity, including TNF, IL1B, IFNG, IL1A, NFkB, TLR7, RELA, interferon alpha, CD40LG, IL27, CSF2, CD40, and TICAM-1. RELA encodes the REL-associated protein that is involved in NF-kB activation^15^. KLF6 (a transcription factor), is induced in early-intermediate hepatic stellate cell activation, consistent with inflammation induced hepatic stellate cell activation^16^. CD40 is a transmembrane protein found on antigen-presenting cells, including macrophages, and upregulation is associated with inflammation and infection^17^. Activation of CD40 (by the CD40 ligand [CD40LG] expressed on activated CD4^+^ T cells) triggers downstream activation of humoral and cellular immunity pathways^18^. Predicted activated upstream regulators that explain downstream changes in gene expression in a disease state are potential targets for inhibitors.

**Table 1:** Top 20 predicted activated and inhibited upstream regulators in Ingenuity Pathway Analysis.

Upstream regulator	Expr log ratio	Molecule type	Activation z-score	p-value of overlap	# genes in network	# regulators in network
TNF		Cytokine	5.247	4.74E−34	235	13
IL1B		Cytokine	5.197	1.87E−21	233	14
IFNG		Cytokine	5.116	1.71E−17	233	15
IL1A		Cytokine	4.918	5.42E−16	219	16
KLF6	1.063	Transcription regulator	4.697	1.31E−12	179	17
NFkB (complex)		Complex	4.357	4.17E−24	254	15
CDKN2A		Transcription regulator	4.316	3.01E−11	251	23
TLR7		Transmembrane receptor	4.042	1.63E−07	219	17
RELA	0.372	Transcription regulator	3.722	3.63E−12	261	15
Interferon alpha		Group	3.487	1.85E−11	176	15
SMARCB1	0.068	Transcription regulator	3.487	1.44E−07	222	18
ZFTA-RELA		Fusion gene/product	3.464	8.16E−09		
BHLHE40	-0.027	Transcription regulator	3.453	7.06E−07	191	12
CD40LG	0.018	Cytokine	3.447	4.85E−12	230	14
LDL		Complex	3.404	4.42E−06	281	20
IL27	0.617	Cytokine	3.368	3.9E−09	253	17
CSF2		Cytokine	3.367	1.39E−12	238	14
CD40	1.287	Transmembrane receptor	3.336	3.35E−07	211	13
ZBTB10	0.309	Transcription regulator	3.317	0.00506	109	7
TICAM1	0.211	Other	3.303	1.14E−05	197	20
E2F1	− 1.819	Transcription regulator	− 4.415	4.48E−12	162	15
E2f		Group	− 3.925	2.41E−13	142	9
CD3E	− 0.188	Transmembrane receptor	− 3.873	1.06E−12	224	19
CITED2	− 0.162	Transcription regulator	− 3.786	9.99E−06	196	14
TREX1	0.541	Enzyme	− 3.721	1.03E−09	145	12
ACOX1	− 0.034	Enzyme	− 3.441	1.09E−08	176	7
IL1RN		Cytokine	− 3.373	1.36E−09	237	17
RNASEH2B	0.045	Other	− 3.357	1.05E−07		
Ttc39aos1		Other	− 3.284	2.48E−06	128	7
Eldr		Other	− 3.162	5.85E−06		
GFI1	2.419	Transcription regulator	− 3.162	0.00127	211	13
ZBTB20	− 0.426	Transcription regulator	− 3.148	2.94E−06	136	7
SP110	0.31	Transcription regulator	− 3.051	0.00031		
ABCB4		Transporter	− 2.996	2.23E−08	118	7
EP400	0.358	Other	− 2.985	4.47E−08		
TARDBP	0.083	Transcription regulator	− 2.764	4.57E−05		
E2F3	0.137	Transcription regulator	− 2.72	2.71E−09	164	16
miR-199a-5p		Mature microrna	− 2.714	2.9E−06		
SSTR2		G-protein coupled receptor	− 2.714	1.6E−08	251	23
TNFRSF9	3.908	Transmembrane receptor	− 2.692	3.69E−12	171	15

Drugs and other chemicals are not included.

### Potential therapeutics

**Figure 8:**
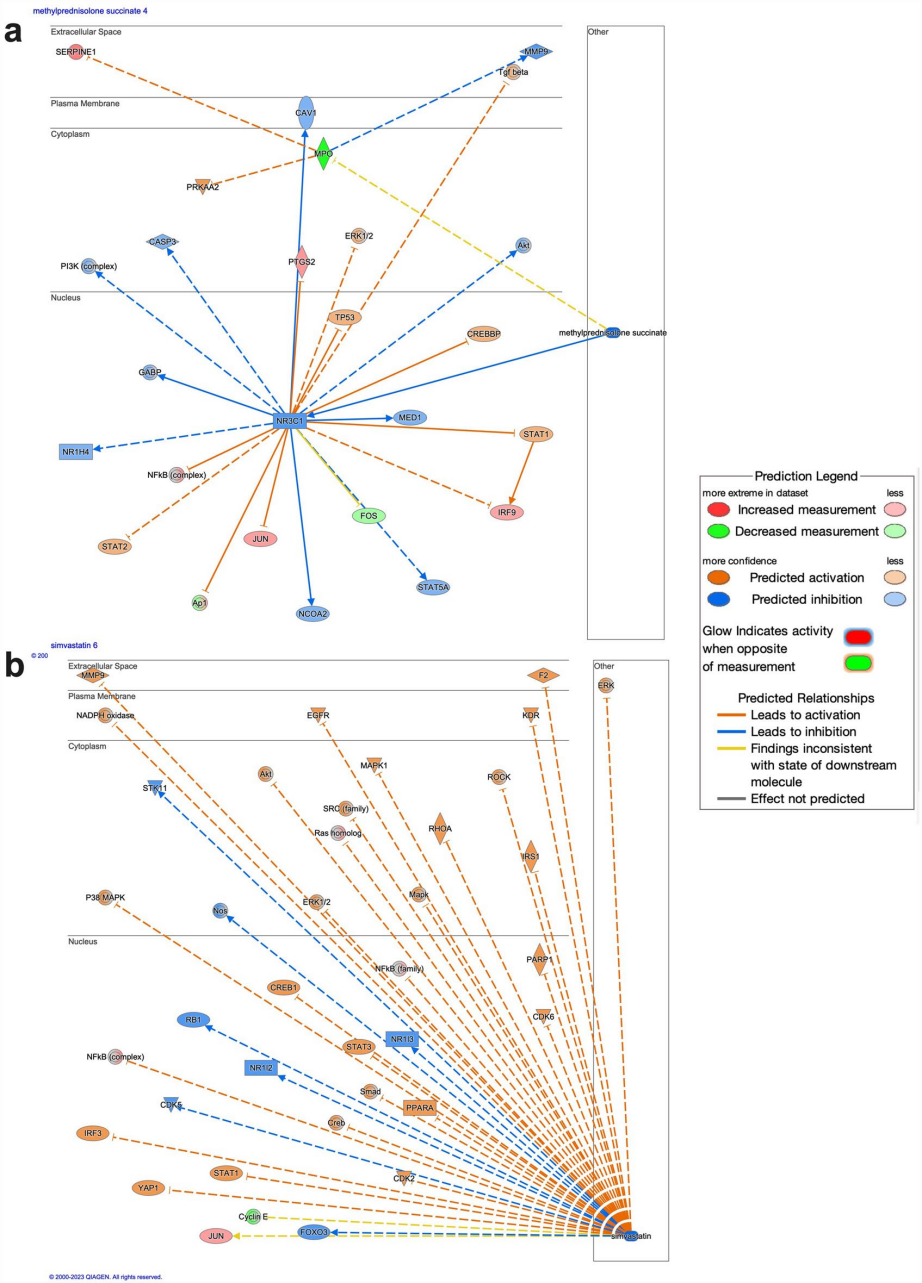
Potential therapeutics identified as strongly inhibited upstream regulators in Ingenuity Pathway Analysis. Methylprednisolone succinate is predicted to directly counteract multiple upregulated inflammatory regulators including NF-kB, STAT2, IRF9, and JUN via NR3C1 (**a**). Simvastatin is predicted to indirectly inhibit multiple inflammatory regulators including NF-kB, STAT1, STAT3, Smad, and JUN (**b**). Darker color demonstrates greater magnitude of pathway activation or inhibition.

Several drugs were identified as strongly inhibited upstream master regulators on causal network analysis in Ingenuity Pathway Analysis (Table 2). Treatment with these drugs may counteract the pathologic changes in gene expression from the disease state. Master regulators are upstream from multiple regulators, which then further influence downstream pathways. For example, methylprednisolone succinate, a corticosteroid, is predicted to counteract the activation of key acute phase response genes including NF-kB, STAT1, STAT2, JUN, FOS, TGFB, NR1H4, PI3K, and CASP3 via the glucocorticoid receptor NR3C1 (Fig. 8a). Additional drugs that act directly on the immune system include another glucocorticoid (betamethasone valerate), an anti-TNF monoclonal antibody (infliximab), a cyclooxegenase-2 inhibitor (SC-58125), and an NF-kB inhibitor (IKK-2 inhibitor VIII). Obeticholic acid, a semi-synthetic bile acid analogue, was also identified as a potential therapeutic via inhibition of NF-kB and activation of NR1H4 (FXR). Outside of the top 20 inhibited master regulators was simvastatin (33rd highest, z-score: -3.018), which has a broad set of downstream anti-inflammatory and metabolic effects via inhibition of NFkB, STAT1, STAT3, and Smad, among others (Fig. 8b).

**Table 2:** Top 20 biologic and chemical drugs identified as inhibited master regulators by causal network analysis in ingenuity pathway analysis.

Upstream regulator	Molecule type	Activation z-score	p-value of overlap	# genes in network	# regulators in network
Dienogest	Chemical drug	− 5.488	8.26E−28	202	40
Paricalcitol	Chemical drug	− 4.911	4.25E−28	209	51
Infliximab	Biologic drug	− 4.667	1.21E−32	144	3
SC-58125	Chemical drug	− 4.469	3.66E−32	146	4
Bosentan	Chemical drug	− 4.216	2.39E−30	90	9
CGP53716	Chemical drug	− 4.041	2.61E−29	75	2
Dicumarol	Chemical drug	− 4.025	1.18E−25	80	4
Erythromycin	Chemical drug	− 3.976	2.52E−24	82	8
Ambroxol	Chemical drug	− 3.75	2.49E−24	64	4
Obeticholic acid	Chemical drug	− 3.671	6.42E−28	76	4
Glycyrrhizic acid	Chemical drug	− 3.624	4.22E−26	88	10
Trimetazidine	Chemical drug	− 3.578	1.43E−25	80	7
IKK-2 inhibitor VIII	Chemical drug	− 3.464	1.9E−37	192	33
Methylprednisolone succinate	Chemical drug	− 3.41	4.31E−29	159	26
Betamethasone valerate	Chemical drug	− 3.41	4.31E−29	159	26
Milrinone	Chemical drug	− 3.394	2.32E−24	73	5
Rottlerin	Chemical drug	− 3.359	1.22E−27	149	25
KPT-9274	Chemical drug	− 3.359	7.94E−32	149	21
Acamprosate	Chemical drug	− 3.302	1.02E−30	106	12
Damnacanthal	Chemical drug	− 3.273	5.5E−27	84	4

## Discussion

This is the first investigation, to our knowledge, that has used RNA-Seq in a large animal model of IFALD to elucidate the early molecular basis of the disease and identify potential therapeutics. The preterm piglet model of IFALD is ideally suited for mechanistic study, as the pathogenesis of liver disease is dependent upon (1) prematurity, resulting in an immature liver susceptible to oxidative damage, and (2) provision of PN with a soybean oil lipid emulsion with similar composition to human neonates. These factors, as in human premature neonates, result in an active IFALD phenotype comprising cholestatic liver injury (elevations in direct bilirubin, total bilirubin, gamma glutamyl transferase) even in the absence of bowel resection or sepsis^10^. Swine also have remarkably similar gastrointestinal anatomy and physiology to humans, making them an ideal model organism for translational research^19^. Utilizing liver tissue from preterm piglets that received either two weeks of PN or sow-milk replacer, we demonstrate that early IFALD is primarily an inflammatory liver disease with broad-based activation of inflammatory pathways including TNF and NF-kB signaling, which ultimately drives the progression to cholestasis and fibrosis. Thus, preventing the development and progression of IFALD may depend upon modulating this inflammation.

Previous investigations have identified numerous risk factors for development of IFALD^5^. The common thread between these factors is unopposed pro-inflammatory insults (bacterial product translocation into the portal circulation, sepsis, pro-inflammatory lipids and phytosterols) with disruption of the enterohepatic axis. Thus, hepatoprotective management has primarily involved the avoidance of toxicity (e.g., preventing central venous catheter infections, lipid dose reduction, switching to more omega-3 rich lipids)^20^. In fact, the most successful treatment for IFALD is pure fish oil lipid emulsion, which replaces the standard soybean oil lipid emulsion–rich in pro-inflammatory omega-6 fatty acids and phytosterols–with an omega-3 rich lipid emulsion with essentially no phytosterol content^3^. Early IFALD has not been studied on a mechanistic level in humans, as liver biopsies are not routinely performed in this population. Previous work found that developing human livers, compared to adult livers, have downregulated xenobiotic, bile acid, and fatty acid metabolism–which may increase susceptibility to PN-induced liver injury^21^. One previous study has used RNA-Seq in a rat model of IFALD and found the primary perturbations were in metabolic pathways (e.g. AMPK signaling, PPAR signaling) and bile acid transport/synthesis, rather than inflammatory pathways^6^. A second study used a microarray analysis in a murine model of IFALD, finding that many of the more enriched KEGG pathways were inflammatory (e.g. cytokine-cytokine receptor interaction, Jak-STAT signaling pathway, natural killer cell-mediated cytotoxicity), with lesser enrichment (but no consistent up/downregulation) of genes in metabolic and bile homeostasis pathways^7^. These results are consistent with our RNA-Seq analysis, highlighting that early IFALD is primarily an inflammatory disease with secondary effects on metabolism and bile acid homeostasis.

Our analysis here identified several additional therapeutics that may reverse the inflammatory transcriptomic phenotype, including therapies that directly inhibit components of the inflammatory response such as infliximab (anti-TNF monoclonal antibody), IKK-2 Inhibitor VIII (anti-NF-kB), methylprednisolone succinate (synthetic glucocorticoid with broad anti-inflammatory effects), and simvastatin, which affects a broad array of metabolic and inflammatory pathways. Infliximab has been studied in a murine model of parenteral nutrition-associated liver disease. Treatment with infliximab decreased biochemical and histologic cholestasis, while increasing transcription of bile acid transporters^22^. In humans, infliximab has been investigated as a rescue therapy for autoimmune hepatitis, although disappointing results were seen in trials of primary sclerosing cholangitis with concurrent inflammatory bowel disease, as well as mixed results for alcoholic hepatitis^23–26^. A key concern with targeting components of the inflammatory response is the risk of immunosuppression and opportunistic infection. Infliximab has boxed warnings for serious infections and malignancy^27^. These infections include opportunistic invasive fungal and bacterial infections. For patients with IFALD, who are already at high risk for sepsis from indwelling central venous catheters and gut bacterial translocation, this may lead to prohibitively high infectious risk. Further, children treated with infliximab and other anti-TNF therapies have developed lymphoma and other malignancies^27^. For the neonate or child with IFALD, these limitations likely preclude use of infliximab or other anti-TNF therapies.

Methylprednisolone or other glucocorticoid treatment for early IFALD is intriguing. Glucocorticoids broadly suppress the immune response via a combination of inducing anti-inflammatory gene transcription, inhibiting transcription of pro-inflammatory genes and cytokines via transrepression of NF-kB and other transcription factors, and posttranslational mechanisms^28^. The infection risk imparted by glucocorticoid therapy tends to be directly correlated to dosage, allowing for more managable immunosuppression than anti-TNF biologic therapy allows^29^. Glucocorticoid therapy (combined with azathioprine) is the standard treatment for autoimmune hepatitis, with a substantial survival benefit^30^. In alcohol-related acute-on-chronic liver failure, glucocorticoid treatment is recommended based upon improved 28-day mortality^31^. In infants with biliary atresia, glucocorticoid therapy is often utilized for both anti-inflammatory and choleretic effects that may improve the clearance of jaundice—of particular interest for the neonate with active inflammatory and cholestatic IFALD^32^. An intriguing small retrospective cohort study from a single center in China found that late postnatal dexamethasone administration for bronchopulmonary dysplasia in preterm infants was strongly protective against development of parenteral nutrition-associated cholestasis^33^. The established use of glucocorticoids for other inflammatory liver diseases, as well as the plausible biological mechanism as a therapeutic for IFALD, warrants further investigation.

Statin therapy has been investigated extensively in NAFLD and nonalcoholic steatohepatitis (NASH). In animal models of NAFLD/NASH, simvastatin reduces liver inflammation and fibrosis, and suppresses hepatic stellate cell activation^34,35^. In a murine model of NASH, atorvastatin inhibited intestinal apical sodium-dependent bile salt transporter-mediated reabsorption of bile acids, stearoyl-coenzyme A desaturase-1 (key lipogenesis gene), and NF-kB signaling^36^. Indirect PPARα induction by statin treatment with increased fatty acid oxidation is a proposed mechanism of hepatoprotection against NASH^37,38^. In a separate rat model of nonalcoholic fatty liver disease (NAFLD), atorvastatin had antioxidant and anti-inflammatory effects, preventing development of biochemical and histologic disease^39^. Similarly, in a doxorubicin-induced hepatotoxicity model in rats, atorvastatin prevented oxidative stress, inhibited NF-kB and IL-1 signaling, and accelerated hepatic lipid metabolism^40^. Atorvastatin also reduced fibrosis in a rat carbon tetrachloride model of liver fibrosis^41^. In humans, statin use is associated with dose-dependent protection against steatosis, nonalcoholic steatohepatitis (NASH), and fibrosis^42^. A randomized controlled trial of military personnel with NAFLD/NASH found that statin initiation (atorvastatin, rosuvastatin, or pitavastatin, compared to diet and exercise alone) reduced the NAFLD activity score and fibrosis-4 score^43^. Given the extent of preclinical and clinical data supporting statin therapy (particularly atorvastatin) in NAFLD/NASH, as well as a well-established safety profile, statin therapy is another strong candidate for investigation in IFALD.

Obeticholic acid is a synthetic bile acid analogue of chenodeoxycholic acid that acts as a farsenoid X receptor (FXR) ligand, impacting bile acid homeostasis, inflammation, and metabolism in the liver^44^. In patients with primary biliary cholangitis, there is a long-term survival benefit in multiple trials^45,46^. Obeticholic acid and other FXR agonists have been investigated for NAFLD/NASH across several clinical trials, with modestly improved liver histology and biochemistry, although with significant side effects including drug-induced liver toxicity, dyslipidemia, and severe pruritis that have limited further development for this indication^44^. Along with other FXR agonists, obeticholic acid has had limited investigation in animal models of IFALD and has been previously suggested as a potential therapy for IFALD, although it has not yet been evaluated in humans^5^.

This study had several strengths. In addition to use of a controlled large-animal model with excellent gastrointestinal homology to humans, the disease phenotype is initiated by the provision of PN including a soybean oil lipid emulsion. A strength of RNA-Seq with subsequent analysis of canonical pathways is that it is unbiased, as the entirety of the mRNA transcriptome is assessed (as opposed to evaluating transcription of individual genes of interest with targeted polymerase chain reaction or even microarrays). Similarly, individual transcripts or proteins do not act in isolation, and a better global understanding of cellular function is provided by a pathway-based approach. This agnostic approach allows for a detailed understanding of perturbations in cellular homeostasis, as well as identification of potential therapeutics. However, there are also several limitations. mRNA-Seq can only identify differences in mRNA expression, which may not reflect actual protein expression and function due to silencing and post-translational modification. In this study, bulk RNA sequencing of liver tissue was performed which provides a global understanding of the hepatic transcriptome, but not the role of individual cells in the disease pathogenesis. Future studies may consider single-cell RNA-Seq to expand on the current findings with insights specific to individual cell types.

A key limitation of this study was the small sample size, with three piglets in the control group and five included piglets in the disease group. The small sample size may increase variability and decrease the power to detect important differences in gene expression. However, despite this, the differential gene expression between the two groups was substantial, resulting in 2060 identified DEGs defined by a *P* < 0.05 with multiple testing adjustment. A more stringent criterion was imposed in Ingenuity Pathway Analysis, only assessing molecules with an adjusted *P* < 0.05 and an expression log-fold change > |1|. An additional limitation is that the bottle-fed control piglets were from a separate litter, although they were delivered at the same gestational age from the same pig strain with sows obtained from the same farm. This may have produced confounding that cannot be accounted for in this paper. This model may better represent the active inflammatory and cholestatic disease seen in premature neonates, and the results may not be translatable to the older patient population which often demonstrates a steato-fibrotic phenotype. Finally, while potential therapeutics were identified based upon analysis of DEGs and the potential to reverse the dysregulated inflammatory pathways, these therapeutics will need to be further validated in animal models and ultimately investigated in clinical trials.

### Conclusions

In a pilot, hypothesis-generating study, we report the first evaluation of liver tissue transcriptomics in a large animal model of IFALD that is highly analogous to the preterm neonate. Early IFALD is characterized by broad activation of hepatic inflammatory pathways. We have identified several potential therapeutics with anti-inflammatory effects that may rescue PN-induced gene dysregulation.

## Methods

### Study design

The aim of this study was to investigate the underlying mechanisms of liver injury in early IFALD, utilizing liver tissue obtained from a preterm piglet model of IFALD compared to a second cohort of bottle-fed age-matched preterm piglets as a healthy control in a post hoc analysis. The tissue and transcriptomic data for the PN cohort of piglets have been previously reported in a study of the efficacy of an investigational therapeutic^10^. This study is a secondary use of the existing data for the PN cohort. A second cohort of bottle-fed control piglets is described here and is utilized as a healthy control; this second cohort has not been previously reported. The PN cohort and the bottle-fed control cohort were obtained from separate sows/litters.

All procedures were conducted in accordance with the Guide for the Care and Use of Laboratory Animals and approved by the Boston Children’s Hospital Institutional Animal Care and Use Committee^47^. ARRIVE guidelines were followed. Briefly, two pregnant Yorkshire sows (*Sus scrofa domesticus*) were obtained (Parson’s Farm, Hadley, MA) and stabilized in our facility for five days. Piglets were delivered via caesarean section at 111-days gestation (five days preterm). Piglets were resuscitated and jugular central venous catheters were placed immediately following stabilization.

### Experimental groups

PN was initiated on DOL 1 in all piglets after central venous catheter placement. PN was compounded using premixed PN solution (Clinimix E 8/14, Baxter, Deerfield, IL), soybean oil lipid emulsion (Nutrilipid, B. Braun, Bethlehem, PA), intravenous multivitamins (Infuvite, Baxter, Deerfield, IL), and sterile water for injection (Baxter, Deerfield, IL). Piglets in the PN group were advanced to goal volume over five days. PN was continued until sacrifice at DOL 15. The PN control group was previously reported and piglets received a medium chain triglyceride vehicle (3 mL/kg) daily via orogastric gavage^10^. Beginning on the first night of life, bottle-fed piglets were bottle fed every three hours with commercially available sow milk replacer (Grade A Ultra 24 Multi-Species Milk Replacer, Sav-A-Caf, Inc., Chilton, WI). Piglets in the bottle-fed control group were weaned off PN by DOL 3 as bottle feeds were increased.

### Blood collection and biochemical assessment

Blood was collected via central venous catheters on DOL 1 (prior to initiation of PN), DOL 8, and DOL 15 (immediately prior to sacrifice) into lithium heparin plasma separator tubes (BD Microtainer, Franklin Lakes, NJ, USA). Plasma was obtained by centrifugation at 2000×*g* 15 min at 20 °C. Plasma total bilirubin and direct bilirubin were performed by the Boston Children’s Hospital clinical laboratory. Gamma glutamyl transferase was assessed using the VetScan VS2 (Zoetis, Parsippany, NJ, USA).

### Sacrifice and tissue collection

Piglets were sacrificed with sodium pentobarbital (110 mg/kg) on DOL 15. Liver tissue from the right median lobe was collected at necropsy for RNA-Seq, flash-frozen, and stored at – 80 °C until analysis.

### mRNA-Seq and transcriptomic analysis

mRNA-Seq was performed by Novogene Co. (Sacramento, CA, USA). In agreement with best practices for RNA-Seq experiments, a minimum of three biological replicates were used in each group and all samples were processed in the same batch^48^. RNA integrity was assessed with the RNA Nano 6000 Assay kit (Agilent Technologies, CA, USA). All samples had an RNA integrity number > 4 and passed the established quality thresholds. mRNA was purified using poly-T oligo-attached magnetic beads and library quality was assessed using the Agilent Bioanalyzer 2100 system. RNA-Seq was performed on the Illumina NovaSeq 6000 platform with 150 bp paired-end reads. Raw data were cleaned with removal of low-quality reads and then mapped to the Sscrofa11.1 pig genome using Hisat2 v2.0.5 Read counts were then calculated by featureCounts software (v 2.0.3)^49^. DEGs were identified by using the DESeq2 (v 1.38.3) R package (adjusted *P* < 0.05)^50^. Due to the complexity of performing of RNA-Seq, technical variance may result in outliers. The impact of outliers is compounded by high-dimension data with relatively small sample sizes, contributing to increased variance and decreased power for detecting differences between experimental groups^51^. Standard quality assurance for RNA-Seq analysis includes identification of outliers, typically with a principal component analysis^48,51^. Based upon a principal component analysis and clustering of samples using normalized data, one outlier in the PN group was identified and excluded (Fig. 2). The included animals had a similar sex distribution in each group: there were two male and one female piglet (66% male) in the bottle-fed group, compared to three male and two female piglets (60% male) in the PN group.

### Pathway enrichment analysis

KEGG pathways enrichment analysis was performed by the clusterProfiler (v 4.6.2) R package^11–14^. A pathway was treated as significantly enriched if adjusted *P* < 0.05 (with Benjamini–Hochberg correction). Further functional pathway analysis and investigation of upstream regulators was performed using Ingenuity Pathway Analysis (Qiagen Inc., Redwood City, CA) using differentially expressed genes with adjusted *P* < 0.05 and log2foldchange > |1|^52–55^. The analysis settings included direct and indirect relationships, all node types, all data sources, and all species. The tissues and cell lines selected included all cell types, with organ systems filtered to liver and lymph node, and cell lines restricted to hepatoma and immune cell lines to focus the analysis on the most relevant literature-derived molecular relationships. RNA-Seq data are available in the National Center for Biotechnology Information Gene Expression Omnibus with accession number GSE234108^56^.

### Statistical analysis

Biochemical outcomes were assessed in GraphPad Prism version 10.1.1 (GraphPad Software, Boston, MA) using Welch’s t-test with Holm-Sidak adjustment for multiple comparisons. All assessments of differentially expressed genes and pathways were assessed using adjusted *P* < 0.05 to account for multiple testing within the relevant bioinformatics software packages discussed above. All authors had access to the study data, and have reviewed and approved the final manuscript.

## Data Availability

RNA-Seq data are available in the National Center for Biotechnology Information Gene Expression Omnibus with accession number GSE234108 (https://www.ncbi.nlm.nih.gov/geo/query/acc.cgi?acc=GSE234108)^56^.

## Abbreviations

DOL: Day of life
DEGs: Differentially expressed genes
FXR: Farsenoid X receptor
IFALD: Intestinal failure-associated liver disease
KEGG: Kyoto encyclopedia of genes and genomes
NASH: Nonalcoholic steatohepatitis
NAFLD: Nonalcoholic fatty liver disease
NF-kB: Nuclear factor kappa B
PN: Parenteral nutrition
RNA-Seq: RNA sequencing
TNF: Tumor necrosis factor
TLR: Toll like receptor
